# Is elevated Red cell distribution width a prognostic predictor in adult patients with community acquired Pneumonia?

**DOI:** 10.1186/1471-2334-14-129

**Published:** 2014-03-05

**Authors:** Eyal Braun, Jad Kheir, Tanya Mashiach, Mohammad Naffaa, Zaher S Azzam

**Affiliations:** 1 Departments of Medicine H and B, Rambam Health Care Campus, P.O. Box 9602, 31096 Haifa, Israel; 2 Biostatistics Unit, Rambam Health Care Campus, Haifa, Israel; 3 Rappaport Family Faculty of Medicine, Haifa, Israel; 4 Research Institute. Technion, Israel Institute of Technology, Haifa, Israel

**Keywords:** Community acquired pneumonia, Red blood cell distribution width, Mortality, Complicated hospitalization

## Abstract

**Background:**

Community acquired pneumonia (CAP) is a major cause of morbidity and mortality. We recently demonstrated that among young patients (<60 years old) with CAP, elevated red blood cell distribution width (RDW) level on admission was associated with significant higher rates of mortality and severe morbidity. We aimed to investigate the prognostic predictive value of RDW among CAP patients in general population of internal wards.

**Methods:**

The cohort included patients of 18 years old or older who were diagnosed with CAP (defined as pneumonia identified 48 hours or less from hospitalization) between January 1, 2005 and December 31, 2010. Patients were retrospectively analyzed for risk factors for a primary endpoint of 90-day mortality. Secondary endpoint was defined as complicated hospitalization (defined as at least one of the following: In- hospital mortality, length of stay of at least 10 days or ICU admission). Binary logistic regression analysis was used for the calculation of the odds ratios (OR) and p values in univariate and multivariate analysis to identify association between patient characteristic, 90-day mortality and complicated hospitalization.

**Results:**

The cohort included 3815 patients. In univariate analysis, patients with co-morbid conditions tended to have a complicated course of CAP. In multivariate regression analysis, variables associated with an increased risk of 90-day mortality included age > 70 years, high Charlson comorbidity index (>2), Hb < 10 mg/dl, Na <130 meq/l, blood urea nitrogen (BUN) >30 mg/dl, systolic blood pressure < 90 mmHg and elevated RDW >15%. Variables associated with complicated hospitalization included high Charlson comorbidity index, BUN > 30 mg/dl, hemoglobin < 10 g/dl, heart rate >124 bpm, systolic blood pressure < 90 mmHg and elevated RDW. Mortality rate and complicated hospitalization were significantly higher among patients with increased RDW regardless of the white blood cell count or hemoglobin levels.

**Conclusions:**

Elevated RDW levels on admission are associated with significant higher rates of mortality and severe morbidity in adult patients with CAP. RDW as a prognostic marker was unrelated with hemoglobin levels, WBC count, age or Charlson score.

## Background

Community acquired pneumonia (CAP) is among the leading causes of mortality and severe morbidity especially in elderly population. Despite the efficacy of modern antibiotic treatment, it still ranks as the sixth most common cause of death [1-3]. Prognostic scores, like the CURB65 score and the Pneumonia Patient Outcomes Research Team score, were developed to estimate the risk of adverse outcome in patients treated in emergency rooms in an attempt to determine who is at risk for an adverse outcome, and therefore should be hospitalized [4,5].

Red blood cell distribution width (RDW) is a laboratory test used to evaluate variance in size or form of red blood cell. It is an important marker for the differential diagnosis of microcytic anemia. However, elevated RDW has been recently shown to be associated with adverse prognosis in several cardiac conditions [6-8], as well as acute stroke [9], pulmonary thromboembolism [10], chronic kidney disease [11], and septic shock [12].

The exact mechanisms causing elevated RDW in these diverse conditions are unknown, however, it is assumed to be related to inflammatory processes that might interfere with the process of erythropoesis [13].

We have recently shown that among young patients (60 years age and below), elevated RDW on admission, either alone or in combination with abnormal white blood cell count (WBC) count, was associated with adverse outcome (defined as 90 days mortality and complicated hospitalization) [14] . This prognostic value was unrelated to hemoglobin levels. As young patients are an important, but relatively small portion of patients seen in emergency rooms with CAP, we aimed to investigate whether this association is true also among the general population, especially in the elderly patients.

## Methods

Patients aged 18 years old or older who were diagnosed with CAP (defined as pneumonia identified within the first 48 hours of hospitalization) between 1 March, 2005 and 31 December, 2010 were retrospectively analysed to identify risk factors for complicated hospitalization and 90-day mortality. The study was performed at Rambam Health Care Campus which is a tertiary public government hospital with 1000 admission beds in Northern Israel and serves 1.5 million citizens. Data was collected from the Prometheus, which is an integrated computer system for handling patients’ medical records. The 90-day mortality data was retrieved from the database of our hospital and the ministry of health. We included only patients who had radiologically confirmed new infiltrates.

Complicated hospitalization was defined as at least one of the following parameters: hospitalization longer than 10 days, admission to ICU and in- hospital mortality. Otherwise, the hospitalization was defined as uncomplicated. The Rambam Hospital Institutional Review Board approved the study. The need for informed consent was waived.

Exclusion criteria included age under 18 years, transfer from another hospital, hospitalization during 30 days prior to admission, hospital-acquired pneumonia (defined as pneumonia which was diagnosed more than 48 hours after admission) or partial antibiotic treatment before hospitalization.

The following data were retrieved from the electronic medical records of the patients:

(1) Malignancies: solid tumours, hematologic malignancies. (2) Pulmonary diseases: bronchial asthma, chronic obstructive lung disease, interstitial lung disease, bronchiectasis, permanent tracheostomy, lung malignancy, past history of thoracic radiotherapy, previous episode of pneumonia, and previous or current active smoker. (3) Immune suppression conditions: current chronic corticosteroid treatment, current or recent chemotherapy treatment, carrier of HIV, primary immune deficiency, history of bone marrow transplantation. (4) Cardiovascular diseases. (5) Chronic kidney disease including patients on dialysis. (6) Diabetes mellitus. (7) Liver cirrhosis. (8) Prior neurologic damage. (9) Chronic alcohol use. (10) Intravenous drug abuse. (11) Nursing house residents. The Charlson comorbidity index (a score that predicts the ten-year mortality for a patient who may have a range of comorbid conditions, (a total of 22 conditions). Each condition is assigned a score of 1, 2, 3, or 6, depending on the risk of dying associated with each one. Scores are summed to provide a total score to predict mortality) [15] was calculated based upon this data. In addition, the vital signs (heart rate, systolic blood pressure, body temperature and oxygen saturation) of the patients were recorded on admission.

### Laboratory variables on admission

Serum glucose, creatinine, sodium, hemoglobin, WBC, RDW and blood urea nitrogen (BUN) were measured on admission.

Hemoglobin levels, mean corpuscular volume and RDW were measured on admission and prior to hospital discharge, using the Advia 120 Hematology Analyzer (Siemens Healthcare Diagnostics Deerfield, Illinois, USA). Glucose, BUN and creatinine levels were measured using the “Dimension” (Siemens Healthcare Diagnostics Deerfield, Illinois, USA).

RDW is reported as coefficient of variation (in percent) of red blood cell volume. The normal range for RDW in our laboratory is 11.5 to 14.5%. The correctness of this normal range was confirmed by analyzing RDW data in 17,293 ambulatory subjects who attended the Rambam Center for Preventive Medicine for a medical examination and health counselling. In this group, mean RDW was 13.1% (median 13.0%) with 95% confidence interval (CI) of RDW of 12.0 to 14.4%.

### Statistical analysis

Binary logistic regression analysis was used for the calculation of the odds ratios (OR) with 95% CI and *P* values in univariate analysis to identify association between patient characteristic and 90-day mortality and complicated hospitalization. Multivariate forward stepwise logistic regression was performed to assess the relation between patient characteristics: co-morbidities, laboratory results, and 90-day mortality or complicated hospitalizations.

Variables were selected as candidates for the multivariate analysis on the basis of the level of significance of the univariate association with 90-day mortality and complicated hospitalization (*P* < 0.1). Notably, there was no predilection in choosing RDW or any other variable in the statistical model.

The area under curve (AUC) was used as a measure of model of discrimination. The calibration of the prediction equation was assessed by comparing the observed and expected numbers of 90-day mortality or complicated hospitalization. The calibration of the prediction equation was assessed by comparing the observed and expected numbers of 90-day mortality or complicated hospitalization rate by decile of predicted risk. The Hosmer-Lemeshow goodness-of-fit statistic was calculated. Comparing of patients characteristics from two groups (complicated and uncomplicated) was done by using chi-square test. Student’s *t*-test was used to compare age between the two groups, whereas, length of stay was compared using Mann–Whitney nonparametric test. Two-tailed *P* values of 0.05 or less were considered as statistically significant. We calculated the Spearman’s rank correlation coefficient to try to find out correlation between variables that were found positive in the multivariate analysis. All statistical analyses were performed using SPSS (Statistics Products Solutions Services; Armonk, New York, USA) 17.0 software for Windows; Redmond, Washington, USA.

## Results

The cohort included 3815 patients; 56.4% were males, median age was 69.6 years, the in-hospital mortality rate was 14.3% and the median length of stay was six days. The median length of stay was 6 and 18.6 days in uncomplicated and complicated patients, respectively. In patients who had a complicated course of pneumonia, 90-day mortality was 63.3% as compared with 11.6% in uncomplicated patients (*P* < 0.03).

### Univariate analysis of complicated hospitalizations and 90-day mortality

As shown in Table 1, 956 patients (28.1%) experienced complicated hospitalization and 937 (24.6%) patients died within 90 days of hospitalization; as expected, older patients and those with co-morbid conditions (higher Charlson score) tended to have a higher rate of both end points.

**Table 1:** Baseline characteristics of the cohort with univariate analysis of risk factors for the detection of 90-day mortality and complicated hospitalization

		**All patients**	**Complicated admissions**				**90 days mortality**			
		**N (%)**	**N (%)**	**P value**	**Odds ratio**	**95% CI**	**N (%)**	**P value**	**Odds ratio**	**95% CI**
		3815	956 (28.1)				937 (24.6)			
**Male (%)**		2153 (56)	564				546 (25.4)		1	-
**Female (%)**		1662 (44)	392	0.07	0.87		391 (23.5)	0.19	0.91	0.78 -1.05
**Age (years)**	<50	592 (16)	98 (16.6)	<0.001	1	1.16-2.19	38 (6.4)	<0.001	1	-
	50–59	395 (10)	95 (24.1)	0.004	1.59	1.05-1.89	61 (15.4)	<0.001	2.663	1.737-4.082
	60–69	573 (15)	125 (21.8)	0.023	1.41	1.3-2.18	101 (17.6)	<0.001	3.12	2.107-4.62
	70–79	971 (25)	243 (25)	<0.001	1.68	1.8-2.99	245 (25.2)	<0.001	4.92	3.435-7.046
	80–89	1004 (26)	316 (31.5)	<0.001	2.32	1.41-2.78	362 (36.1)	<0.001	8.221	5.775-11.701
	≥90	280 (7)	79 (28.2)	<0.001	1.98		130 (46.4)	<0.001	12.635	8.436-18.924
**Charlson scoring**	0	725 (19.0)	92 (12.7)	0.000	1	-	54 (7.4)	.000	1	-
	1	658 (17.2)	127 (19.3)	0.001	1.67	1.24-2.33	120 (18.2)	.000	2.772	1.9-3.8
	2	624 (16.4)	140 (22.4)	0.000	1.98	1.48-2.64	145 (23.2)	.000	3.762	2.6-5.2
	3–4	1002 (26.3)	311 (31.0)	0.000	3.09	2.39-4	310 (30.9)	.000	5.567	4.1-7.5
	5–7	606 (15.9)	214 (35.3)	0.000	3.75	2.85-4.94	216 (35.6)	.000	6.882	4.9-9.5
	8+	200 (5.2)	81 (40.5)	0.000	4.65	3.25-6.65	92 (46.0)	.000	10.585	7.1-15.6

Table 2 shows laboratory parameters checked for association with 90-day mortality and complicated admission.

**Table 2:** Laboratory and hemodynamic characteristics of the cohort with univariate analysis of risk factors for the detection of 90-day mortality and complicated hospitalization

			**Complicated admissions**				**90 days mortality**			**95% CI**
		**N**	**N (%)**	**P value**	**Odds ratio**	**95% CI**	**N (%)**	**P value**	**Odds ratio**	
	Total	3815	956 (25.1)				937 (24.6)			
**BUN mg/dl**	≤30	2762	537 (19.4)	-	1	-	486 (17.6)	-	1	-
	>30	1053	419 (39.8)	0.000	2.74	2.34-3.20	451 (42.8)	<0.001	3.51	3–4.10
**Creatinine mg/dl**	≤1.5	3010	645 (21.4)	-	1	-	631 (21.0)	-	1	-
	>1.5	805	311 (38.6)	0.000	2.31	1.95-2.73	306 (38.0)	<0.001	2.31	1.96-2.73
**Hct**	≥30	3238	749 (23.1)	-	1	-	700 (21.6)	-	1	-
	<30	548	199 (36.3)	0.000	1.90	1.56-2.30	230 (42.0)	<0.001	2.62	2.17-3.17
**RDW %**	≤15	2426	481 (19.8)	-	1	-	408 (16.8)	-	1	-
	>15	1389	475 (34.2)	0.000	2.10	1.81-2.44	529 (38.1)	<0.001	3.04	2.61-3.54
**Glucose mg/dL**	≤250	3491	843 (24.1)	-	1	-	838 (24.0)	-	1	-
	>250	293	107 (36.5)	0.000	1.81	1.41-2.32	94 (32.1)	0.002	1.50	1.16-1.93
**Hb g/dL**	≥10	3189	726 (22.8)	-	1	-	671 (21.0)	-	1	-
	<10	615	226 (36.7)	0.000	1.97	1.64-2.37	264 (42.9)	<0.001	2.82	2.36-3.82
**Na Meq/L**	<130	393	119 (30.3)	0.000	-	-	111 (28.2)	<0.001	-	-
	130 < <150	3301	782 (23.7)	0.004	0.72	0.57-0.90	753 (22.8)	0.016	0.75	0.59-.0.95
	≥150	88	48 (54.5)	0.000	2.76	1.72-4.43	67 (76.1)	<0.001	8.11	4.74-13.87
**WBC×10**^**9**^**/L**	<4- > 12	2023	576 (28.5)	0.000	1.49	1.28-1.73	574 (28.4)	<0.001	1.56	1.34-1.81
	4 ≤ ≤12	1781	376 (21.1)	-	1	-	361 (20.3)	-	1	-
**PH**	≥7.35	153	37 (24.2)	-	1	-	43 (28.1)	-	1	-
	<7.35	2156	588 (27.3)	0.406	1.18	0.80-1.72	597 (27.7)	0.912	0.98	0.68-1.41
**SBP (mmHg)**	≥90	2858	657 (23.0)	-	1	-	888 (23.9)	-	1	-
	<90	101	54 (53.5)	0.000	3.85	2.58-5.75	49 (48.5)	0.457	1.07	0.89-1.28
**Temparature °C**	<40	2859	665 (23.3)	-	1	-	932 (24.5)	-	1	-
	≥40	17	9 (52.2)	0.642	3.71	1.41-9.64	5 (29.4)	0.642	1.28	0.45-3.65
**Pulse min**^**−1**^	≤124	2791	636 (22.8)	-	1	-	888 (24.0)	-	1	-
	>124	141	60 (42.2)	0.005	2.51	1.78-3.55	49 (35.0)	0.005	1.67	1.17-2.38
**SAT %**	92-100	2041	431 (21.1)	-	1	-	460 (23.0)	-	1	-
	<91	606	190 (31.4)	<0.001	1.71	1.39-2.09	196 (32.0)	<0.001	1.64	1.35-2.01

WBC White Blood Cells; RDW Red Cell Width Distribution.

Hct Hematocrit; Hb Hemoglobin; Na Sodium; SBP Systolic blood pressure; SAT Saturation.

BUN Blood Urea Nitrogen; SAT Oxygen Saturation.

### Multivariate analysis of 90-day mortality

All variables that were found to be associated (*P* < 0.1) with 90-day mortality in the univariate analysis were included in the initial multivariate prediction rule. Results are presented in Table 3. In the model which does not include RDW, variables that were associated with 90- day mortality included: age > 70 years, charlson score >2, systolic pressure < 90 mmHg, BUN > 30 mg/dl, Hb < 10 mg/dl, Na <130 meq/l. Whenever RDW was added to the model, it was associated with 90- day mortality. The model with included RDW improved AUC_ROC_ as compared with a model without RDW from 0.773 (95% CI = 0.756-0.8) to 0.785 (95% CI = 0.769-0.8). The Hosmer-Lemeshev goodness-of-fit statistic across decile of risk was not statistically significant indicating little departure and a perfect fit in both models.

**Table 3:** Results of multivariate analysis of risk factors for 90-day mortality

**Morality**											
		**Model without RDW**					**Model with RDW**				
		**COEF**	**P value**	**Adjusted**	**95% CI for ODDS**		**COEF**	**P value**	**Adjusted**	**95% CI for ODDS**	
				**ODDS**	**Lower**	**Upper**			**ODDS**	**Lower**	**Upper**
Charlson score	0		.000					.000			
	1	0.6	.003	1.7	1.2	2.5	.529	.005	1.7	1.2	2.5
	2	0.7	.000	2.1	1.4	2.9	.626	.001	1.9	1.3	2.7
	3-4	0.9	.000	2.5	1.8	3.5	.806	.000	2.2	1.6	3.2
	5-7	1.1	.000	3.0	2.1	4.3	.971	.000	2.6	1.8	3.8
	8+	1.7	.000	5.7	3.7	8.7	1.550	.000	4.7	3.0	7.3
BUN (mg/dL)	>30	0.5	.000	1.7	1.4	2.0	.518	.000	1.7	1.4	2.0
Hemoglobin (g/dL)	<10	0.9	.000	2.4	2.0	2.9	.667	.000	1.9	1.6	2.4
WBC × 10^9^/L	<4- > 12	0.4	.000	1.4	1.2	1.7	.358	.000	1.4	1.2	1.7
Age Groups (years)	<50		.000					.000			
	50-60	0.5	.018	1.7	1.1	2.7	.532	.022	1.7	1.1	2.7
	60-70	0.5	.014	1.7	1.1	2.6	.498	.022	1.6	1.1	2.5
	70-80	0.9	.000	1.7	1.1	3.6	.855	.000	4.1	2.8	6.1
	80-90	1.4	.000	4.2	2.9	6.2	1.420	.000	4.1	2.8	6.1
	≥90	1.9	.000	6.9	4.5	10.7	1.932	.000	6.9	4.4	10.7
Na (mMol/L)	≥150	1.8	.000	5.8	3.4	9.8	1.785	.000	6.0	3.5	10.1
SBP (mmHg)	<90	0.9	.000	2.5	1.6	3.9	.803	.001	2.2	1.4	3.5
HR (min^−1^)	>124	0.7	.001	1.9	1.3	2.9	.578	.006	1.8	1.2	2.7
O_2_ Saturation	≤91	0.3	.005	1.3	1.1	1.7	.286	.008	1.3	1.1	1.6
RDW	>15						.739	.000	2.1	1.8	2.5
	Constant	−3.6	.000	0.0			−3.777	.000	0.0		

WBC White Blood Cells; RDW Red Cell Width Distribution; Hct Hematocrit; Hb Hemoglobin; Na Sodium; SBP Systolic blood pressure; SAT Saturation; BUN Blood Urea Nitrogen; HR Heart Rate; SAT Oxygen Saturation.

### Multivariate analysis of complicated hospitalizations

All variables that were associated (*P* < 0.1) with complicated hospitalization were included in the initial multivariate prediction rule. Results of multivariate analysis are presented in Table 4.

**Table 4:** Results of multivariate analysis of risk factors for complicated hospitalization

**Complicated admissions**											
		**Model WO RDW**					**Model WITH RDW**				
		**COEF**	**P value**	**Adjusted**	**95% for ODDS**		**COEF**	**P value**	**Adjusted**	**95% for ODDS**	
				**ODDS**	**Lower**	**Upper**			**ODDS**	**Lower**	**Upper**
Charlson Score	0	.000						.000			
	1	0.4	.015	1.5	1.1	2.0	.356	.025	1.4	1.0	1.9
	2	0.5	.003	1.6	1.2	2.2	.399	.013	1.5	1.1	2.0
	3-4	0.8	.000	2.2	1.7	3.0	.723	.000	2.1	1.5	2.8
	5-7	0.9	.000	2.6	1.9	3.5	.854	.000	2.3	1.7	3.2
	8+	1.2	.000	3.3	2.2	4.9	1.070	.000	2.9	2.0	4.3
BUN (mg/dL)	>30	0.6	.000	1.8	1.5	2.1	.586	.000	1.8	1.5	2.1
Hemoglobin (g/dL)	<10	0.5	.000	1.6	1.3	1.9	.328	.001	1.4	1.1	1.7
WBC × 10^9^/L	<4-2<	0.3	.000	1.4	1.2	1.6	.321	.000	1.4	1.2	1.6
Age groups (years)	<50	-	.026	-	-	-	-	.023	-	-	-
	50-60	0.1	.386	1.2	0.8	1.6	.141	.414	1.2	0.8	1.6
	60-70	−0.2	.313	0.8	0.6	1.2	-.190	.253	0.8	0.6	1.1
	70-80	−0.1	.501	0.9	0.7	1.2	-.129	.398	0.9	0.7	1.2
	80-89	0.2	.188	1.2	0.9	1.6	.174	.243	1.2	0.9	1.6
	≥90	0.0	.962	1.0	0.7	1.5	-.013	.946	1.0	0.7	1.4
Na (mMol/L)	≥150	0.8	.001	2.2	1.4	3.4	.763	.001	2.1	1.4	3.4
SBP (mmHg)	<90	1.0	.000	2.9	1.9	4.4	.983	.000	2.7	1.7	4.1
HR (min^−1^)	>124	0.8	.000	2.1	1.5	3.1	.716	.000	2.0	1.4	3.0
O_2_ Saturation	≤91	0.2	.025	1.3	1.0	1.5	.220	.033	1.2	1.0	1.5
Temparature °C	≥40	1.4	.008	3.9	1.4	10.7	1.297	.012	3.7	1.	10.0
RDW	>15						.413	.000	1.5	1.3	1.8
	Constant	−2.3	.000	0.1			−2.371	.000	0.1		

WBC White Blood Cells; RDW Red Cell Width Distribution; Hb Hemoglobin; Na Sodium; SBP Systolic blood pressure; SAT Saturation; BUN Blood Urea Nitrogen; HR Heart Rate; SAT Oxygen Saturation.

The variables that were associated with complicated hospitalization included charlson score > 2, systolic blood pressure <90 mmHg, heart rate >124 bpm, BUN >30 mg/dl and Hb <10 mg/dl. Whenever RDW was added to the model, it was associated with complicated hospitalization. A model with RDW improved AUC_ROC_ as compared with a model without RDW from 0.744 (95% CI = 0.726-0.76) to 0.757 (95% CI = 0.74-0.77). The Hosmer-Lemeshev goodness-of-fit statistic across decile of risk was not statistically significant indicating little departure and a perfect fit in both models.

Spearman Rank Correlation coefficient was used to find whether the parameters that were found to be statistically significant in multivariate analysis, were related to each other. As shown in Table 5, there was no correlation between elevated RDW and other parameters, meaning that RDW represents an independent risk factor for 90-day mortality and complicated hospitalization.

**Table 5:** Spearman rank correlation coefficient parameters

		**Total**	**RDW < 13.2**	**RDW 13.2-15**	**RDW > 15**	**Spearman coefficient**	
		**N**	**N (%)**	**N (%)**	**N (%)**		**P**
Hemoglobin	≥10	3189	456 (17)	1772 (55.6)	961 (30.1)	0.29	<0.001
	<10	615	14.3 (2.8)	176 (28.6)	422 (68.6)		
WBC	<4	133	13 (9.8)	32 (24.1)	88 (66.2)	−0.05	0.001
	≥12	1781	239 (13.4)	920 (51.7)	622 (34.9)		
	4 ≤ ≤12	1890	221 (11.7)	996 (52.7)	673 (35.6)		
Creatinine	<1.5	3010	443 (14.7)	1563 (51.9)	1004 (33.4)	0.16	<0.001
	1.5-2	378	18 (4.8)	192 (50.8)	168 (44.4)		
	>2	409	10 (2.4)	191 (46.7)	208 (50.9)		
Na	<130	393	57 (14.5)	183 (46.6)	153 (38.9)	0.01	0.5
	130 < <150	3301	412 (12.5)	1710 (51.8)	1179 (35.7)		
	≥150	88	4 (4.5)	43 (48.9)	41 (46.6)		
BUN	≤30	2762	420 (15.2)	1452 (52.6)	890 (32.2)	0.17	<0.001
	>30	1019	52 (5.1)	483 (47.4)	484 (47.5)		
HCT	≥30	3238	456 (14.1)	1760 (54.4)	1022 (31.6)	0.24	<0.001
	<30	548	17 (3.1)	180 (32.8)	351 (64.1)		
O_2_ SAT%	92-100	2041	270 (13.2)	1036 (50.8)	735 (36.0)		
	**≤**91	606	54 (8.9)	297 (49.0)	255 (42.1)		
Age	<50	592	174 (29.4)	297 (50.2)	121 (20.4)	0.18	<0.001
	50–59	395	70 (17.7)	204 (51.6)	121 (30.6)		
	60–69	572	67 (11.7)	284 (49.7)	221 (38.6)		
	70–79	972	81 (8.3)	485 (49.9)	406 (41.8)		
	80–89	1004	63 (6.3)	533 (53.1)	408 (40.6)		
	≥90	280	19 (6.8)	149 (53.2)	112 (40.0)		

WBC White Blood Cells; Hct Hematocrit; Hb Hemoglobin; Na Sodium; SAT Saturation; BUN Blood Urea Nitrogen; SAT Oxygen Saturation.

### The association of RDW, Charlson score and mortality

**Figure 1:**
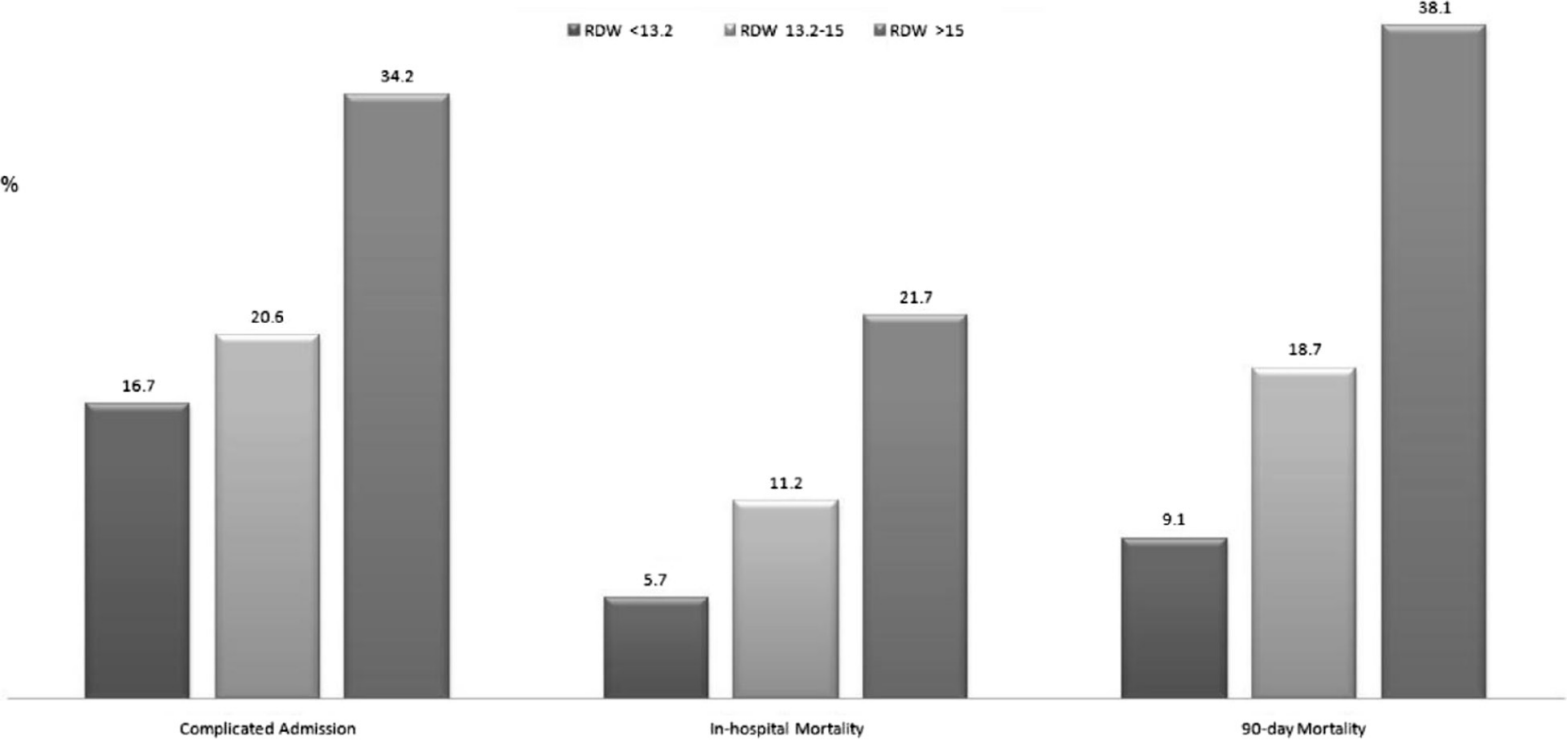
Complicated admission, in-hospital mortality and 90-day mortality association with elevated RDW.

**Figure 2:**
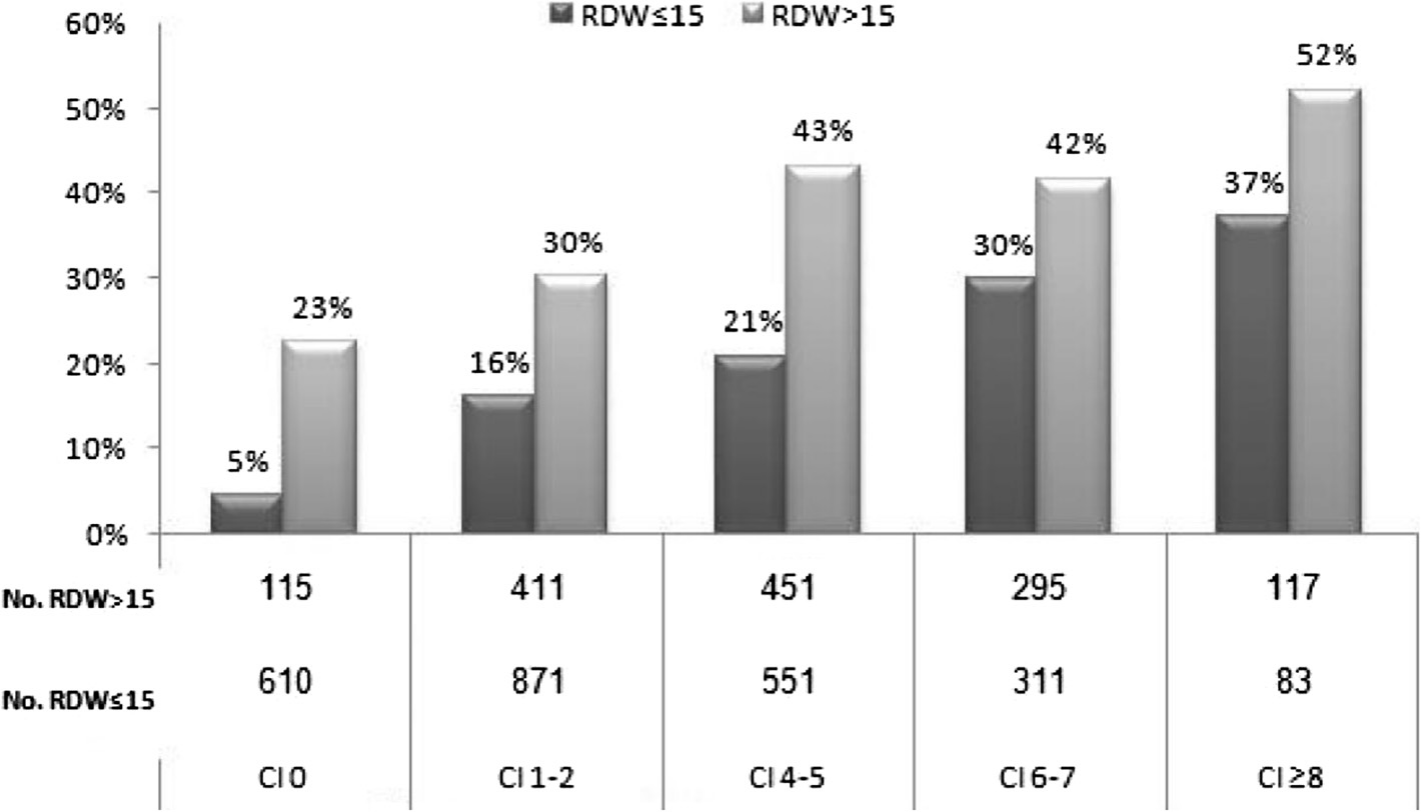
**The relationship between RDW and charlson score.** (CI- Charlson Index).

As depicted in Figure 1, complicated admission, in-hospital mortality and 90-day mortality were directly related with elevated RDW. Notably, higher RDW is correlated with increased mortality, a linkage that was independent with charlson score; even when this score was zero, increased RDW predicted high mortality rates. This relationship was maintained at any charlson score (Figure 2).

### Relation between RDW, age and Charlson score

**Figure 3:**
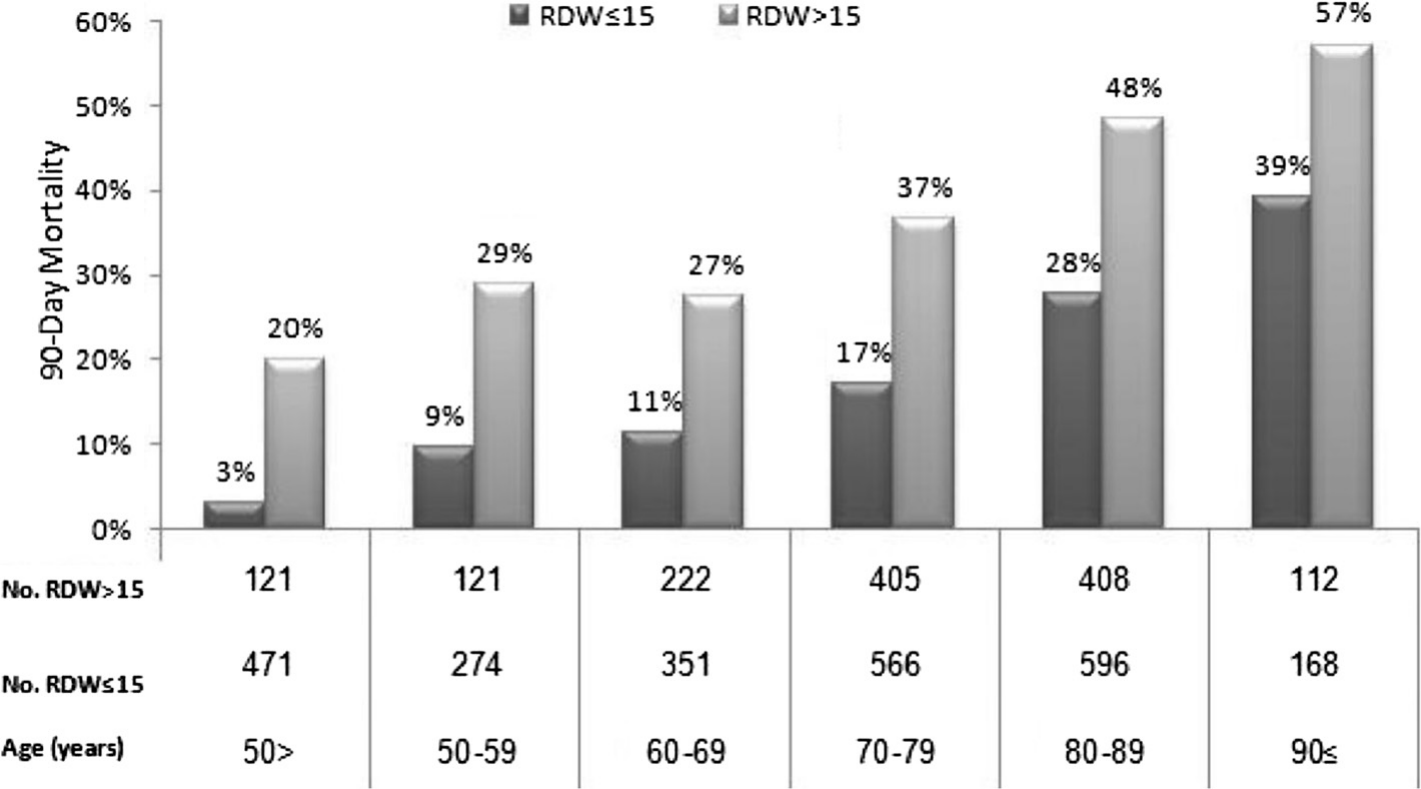
The relation between RDW and age.

As shown in Figure 3, elevated RDW predicted increased mortality rate in all age group. This association was stronger in younger ages (less than 50), by which a 90- day mortality of 20% was observed in patients with RDW >15, as compared to 3% in patients with normal RDW on admission. This relationship was maintained in all age groups.

### The association between the mortality rate, complicated admission with RDW and the different white blood cell groups

**Figure 4:**
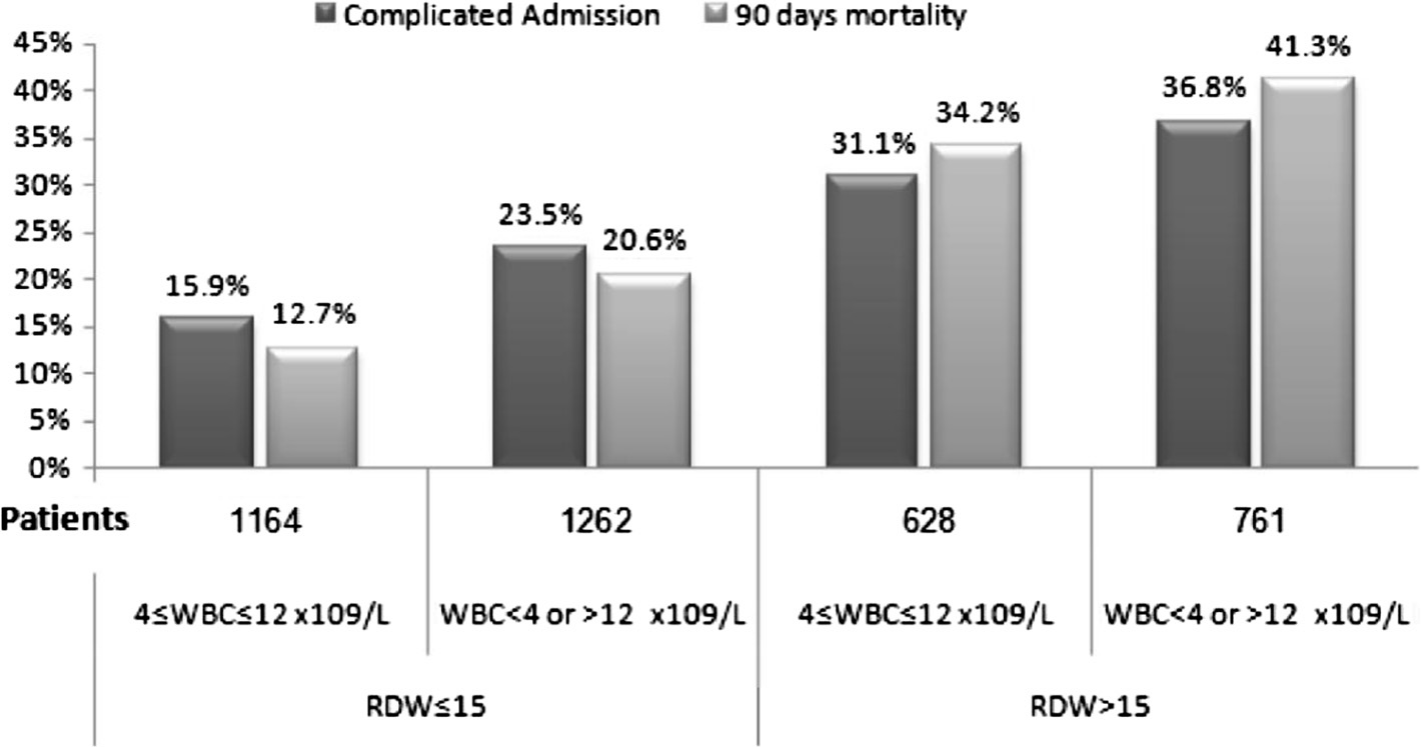
The association between mortality rate, complicated admission with RDW and the different white blood cell groups.

Complicated hospitalization and mortality rates were significantly higher among patients with increased RDW regardless of the WBC count (Figure 4); the complicated hospitalization rate was 15.9% in patients with normal RDW and WBC and 23.5% in patients with only abnormal WBC count, as compared with 31.1% in patients with elevated RDW alone (*P* = 0.001) and 36.8% in patients with combined abnormal leukocyte count and increased RDW (*P* < 0.001). The 90-day mortality was higher in patients with elevated RDW regardless of the WBC count; however, once again the combination of abnormal WBC and elevated RDW was significantly associated with the highest mortality rates.

### The association between the 90-day mortality rate and complicated hospitalization with RDW and hemoglobin groups

**Figure 5:**
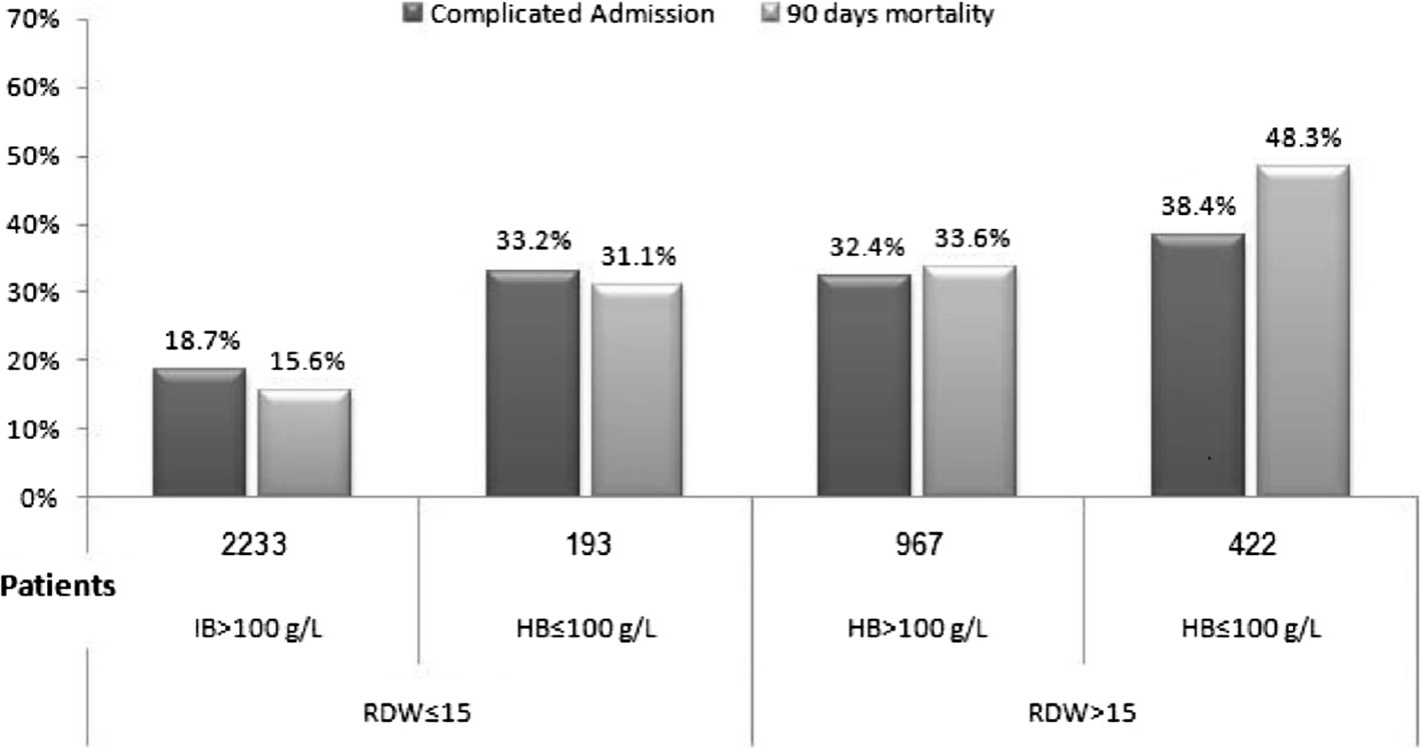
The association between 90-day mortality rate and complicated hospitalization with RDW and different hemoglobin levels.

In order to rule out the possibility that RDW effect on severe morbidity and mortality was related to anemia, we compared complicated hospitalization in patients with hemoglobin levels less than 110 g/L and higher levels of Hb. As depicted in Figure 5, patients with normal RDW had no difference in severe morbidity or mortality regardless of Hb levels. On the other hand, elevated RDW was associated with a significant increase in both complicated hospitalization and 90-day mortality rates irrespective of Hb levels.

### The relationship between adverse outcome, RDW and elevated BUN

**Figure 6:**
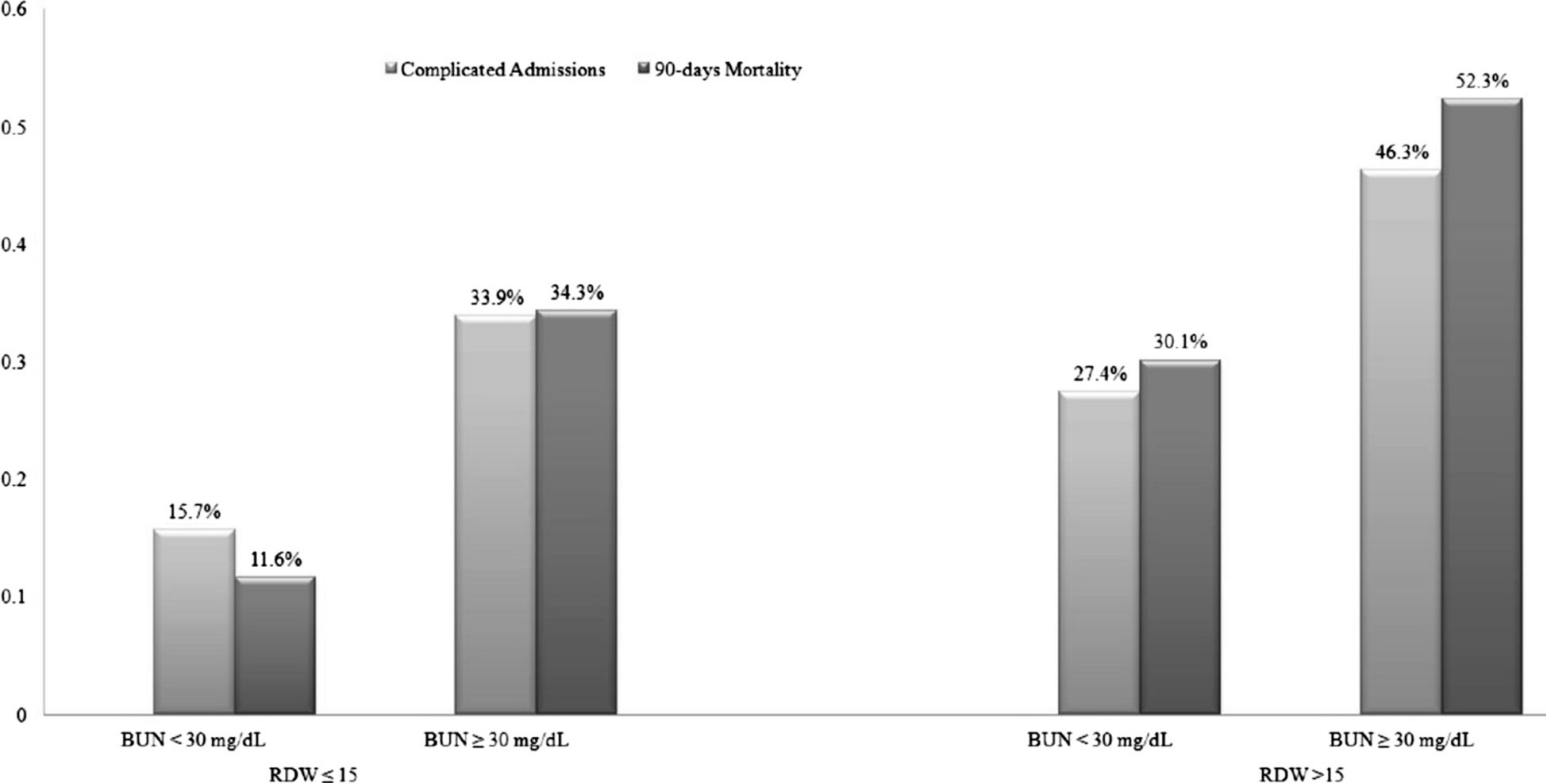
The relationship between RDW, BUN, 90-day mortality and complicated admission.

As shown in Figure 6, combination of elevated BUN and RDW was associated with particularly high rates of primary and secondary endpoints. Notably, more than half of the patients who had high levels of both BUN and RDW on admission died within 90 days.

## Discussion

This study shows that high level of RDW in admission is an independent risk factor for adverse outcome, defined as 90-days mortality and complicated hospitalization. RDW, as a risk factor, was unrelated to other prognostic factors at multivariate analysis.

We previously demonstrated that elevated RDW is associated with significantly higher rates of mortality and severe morbidity among young patients hospitalized with CAP [14]. However, the majority of the patients admitted with CAP are elderly patients, in whom the rates of death and complications are much higher [16]. Despite the progress in antibiotic treatment and handling of critically ill patients in the modern era, the rates of death in this group of patients are still high, making CAP among the leading causes of death in patients in advanced ages. The high burden of CAP and limited number of beds in ICU’s in Israel and many European countries denotes that physicians in the emergency departments need simple and inexpensive tools to decide in a short time frame which patients need hospitalization and particularly who should be a candidate for admission to ICU’s.

Among many risk factors that were examined in this study for adverse outcome in patients with CAP, two stand out: RDW and BUN [17,18]. At ROC models, both parameters were significantly associated with both complicated hospitalization and 90-day mortality compared with other important factors such as elevated glucose, hypernatremia, high levels of creatinine and abnormal WBC counts- all widely considered as adverse factors in CAP and frequently incorporated in various scores (e.g. PORT score) used in flow charts of decision making in emergency departments. Combination of high levels of both RDW and BUN was associated with particularly high levels of mortality; more than half of the patients admitted with high levels of BUN and elevated RDW died within 90 days. Indeed, Hunziker et al. [19] recently suggested that RDW significantly improves risk stratification for simplified acute physiological score to predict short and long term mortality rates in a large and unselected population of ICU patients.

Elevated RDW has been shown to be an important risk factor in various cardiovascular conditions, such as acute decompensated heart failure [20], acute coronary syndromes [6-8] and stroke [9]. Recently, RDW emerged as an independent risk factor in inflammatory and infectious conditions: Ku et al. demonstrated recently that RDW is an independent predictor of mortality among patients with gram-negative bacteremia [21]. Acute and chronic hepatitis B [22] and activity of inflammatory bowel disease [23] have also been recently associated with elevated RDW.

It should be noted that although this cohort included elderly patients in which high rates of anemia of iron deficiency or chronic disease is expected, high rates of adverse outcome were shown in all age and charlson score groups. While this trend is especially notable in younger patients, it is maintained in octogenarian and nanogenerian patients.

In concordance with our study, recently Lee et al. demonstrated in 744 patients that elevated RDW on admission was associated with increased 30-day mortality, length of hospital stay, and use of vasopressors in hospitalized patients with CAP. The inclusion of RDW improved the prognostic performance of the PSI and CURB-65 [24]. Notably, our study included more patients (3815 patients) and showed both a short and long term association with RDW on adult pneumonia outcome. Moreover, we showed that the effect of elevated RDW is maintained in a larger spectrum of age and a variety of co-morbidities.

The mechanism underlying the association between high levels of RDW and adverse outcome in patients hospitalized with pneumonia is unknown. Our data suggests that RDW might be regarded as a valuable and sensitive marker for a high level of inflammatory activity in adult patients with CAP, and it is independent of hemoglobin levels. A mechanism that could be involved in such a process is the release of cytokines in response to inflammatory stress. These cytokines might block the activity of erythropoietin, inhibit erythrocyte maturation and cause production of ineffective red blood cell and elevated RDW [13]. Lippi et al. found a correlation between high RDW and elevated indexes of inflammation, such as elevated erythrocyte sedimentation rate (ESR) and C-reactive protein (CRP). This correlation was independent of concomitant diseases, and was demonstrated even when anemic patients were excluded from the statistical analysis [25]. This might represent a culmination of multiple pathophysiologic processes occurring in acute inflammatory and infectious states. Thus, elevated RDW on admission may indicate a severe inflammatory process, which could be an early sign of adverse prognosis in patients hospitalized with CAP.

The major limitation of this study is that it is retrospective. It should be validated in a prospective study, preferably with inclusion of other inflammatory markers, such as CRP, Procalcitonin and IL-6. Conceivably, RDW should be evaluated for incorporation in the commonly used risk assessment scores. The very old population (>80), included in this study, had high rates of anemia and other pro- inflammatory conditions which might have led to overestimation of RDW as a prognostic factor in this group of patients. Moreover, there were not enough patients in whom iron levels, LDH and other parameters of hemolysis were checked to reach statistical power to include them in the univariate analysis.

## Conclusions

Elevated RDW levels on admission are associated with significant higher rates of mortality and severe morbidity in adult patients with CAP. RDW as a prognostic marker was unrelated with hemoglobin levels or other risk factors for adverse outcome in patients with CAP.

## Competing interests

The authors declare that they have no competing interests.

## Authors’ contributions

EB: conceived of the study, and participated in its design and coordination and drafted the manuscript. JK: participated in data acquisition and drafting manuscript. TM: performed statistical analysis. MN: participated in data acquisition, drafted and revised the manuscript. ZA: conceived of the study, and participated in its design and coordination and drafted the manuscript. All authors read and approved the final manuscript.

## Pre-publication history

The pre-publication history for this paper can be accessed here:

http://www.biomedcentral.com/1471-2334/14/129/prepub
